# Supplementary material for: Carers’ Perspective on Voluntary Stopping of Eating and Drinking: A Systematic Mixed-Methods Review of Motives and Attitudes
Source: Healthcare (Basel). 2025 May 27;13(11):1264. doi: 10.3390/healthcare13111264 (PMC12154388; doi:10.3390/healthcare13111264)
Supplement: Supplementary file 1 [file healthcare-13-01264-s001.zip › healthcare-3589090-supplementary.pdf]

# APPENDIX

## Table of Content


| | |
|---|---|
| 1. Overview of synthesized review findings (master thesis) | p 2 |
| 2. PRISMA 2020 Checklist | p 3 |
| 3. Methodological assessments tables (based on Mixed-Methods Appraisal Tool) | p 5 |
| 4. Evidence Profile Table of the CERQual assessment | p 9 |
| 5. References | p 19 |


Please note, the PRISMA flowchart and the complete search strategy including search terms (text words and MeSH terms) are published in Mensger et al. 2024 and the respective Supplementary material [48].

## 1. Overview of synthesized review findings (master thesis)

Figure S1: Results from the synthesis (master thesis)

**A: VSED in general**
- Relevance of VSED for healthcare professionals (HCPs)
- Is VSED a 'good death'?
- Different forms of VSED - Implicit and concealed VSED

**B: Motives and Decision making**
- Motives: High symptom burden and suffering
- Situation of VSED decision
- Self-determination and autonomy
- VSED as 'best available option' or 'better than other'

**C: Attitudes towards VSED**
- Attitudes of healthcare professionals
- Classification of VSED as 'Natural Dying' or 'Suicide`?

**D: Acceptance of the VSED decision**
- High acceptance of VSED in general
- Acceptance? It also depends...

**E: Support and Accompaniment**
- Accompaniment of VSED and judging the competence by health professionals
- Accompaniment despite concerns
- Support and advocacy by healthcare professionals (HCPs)
- Support is needed
- Support also helps the family caregiver
- Support and advocacy by family caregivers

**F: Challenges for relatives / family caregivers**
- Family members can be burdened / accepting the decision can be difficult
- Legal basis, living will and legitimation
- Managing the care for a loved one and dealing with own feelings
- Issues with professionals and institutions
- The grieving process

**G: Challenges for healthcare professionals**
- Burden, fear and uncertainties among healthcare professionals
- Care for the patient and support of the relatives
- Process in the professional team and in the institution

**H: The needs**
- Knowledge about VSED is important and needed

Eight thematic categories (A - H) with twenty-six review findings. Grey: Review findings presented elsewhere[1]. Dark Grey: Review findings presented in this publication.

## 2. PRISMA Checklist

Table S1: PRISMA 2020 Main Checklist[2]

| Topic | No. | Item | Location where item is reported |
|---|---|---|---|
| TITLE | | | |
| Title | 1 | Identify the report as a systematic review. | **p1** |
| ABSTRACT | | | |
| Abstract | 2 | See the PRISMA 2020 for Abstracts checklist | **p1** |
| INTRODUCTION | | | |
| Rationale | 3 | Describe the rationale for the review in the context of existing knowledge. | **p2** |
| Objectives | 4 | Provide an explicit statement of the objective(s) or question(s) the review addresses. | **p2** |
| METHODS | | | |
| Eligibility criteria | 5 | Specify the inclusion and exclusion criteria for the review and how studies were grouped for the syntheses. | **p3** & Mensger et al. 2024[1] (Table 2) |
| Information sources | 6 | Specify all databases, registers, websites, organisations, reference lists and other sources searched or consulted to identify studies. Specify the date when each source was last searched or consulted. | **p3** & Mensger et al. 2024[1] (Appendix, sec. 4) |
| Search strategy | 7 | Present the full search strategies for all databases, registers and websites, including any filters and limits used. | Mensger et al. 2024[1] (Appendix, sec. 4) |
| Selection process | 8 | Specify the methods used to decide whether a study met the inclusion criteria of the review, including how many reviewers screened each record and each report retrieved, whether they worked independently, and if applicable, details of automation tools used in the process. | Mensger et al. 2024[1] (p5–6) |
| Data collection process | 9 | Specify the methods used to collect data from reports, including how many reviewers collected data from each report, whether they worked independently, any processes for obtaining or confirming data from study investigators, and if applicable, details of automation tools used in the process. | Mensger et al. 2024[1] (p6 & Appendix, sec. 5) |
| Data items | 10a | List and define all outcomes for which data were sought. Specify whether all results that were compatible with each outcome domain in each study were sought (e.g. for all measures, time points, analyses), and if not, the methods used to decide which results to collect. | Mensger et al. 2024[1] (p4 & Table 1, SPIDER structure) |
| | 10b | List and define all other variables for which data were sought (e.g. participant and intervention characteristics, funding sources). Describe any assumptions made about any missing or unclear information. | Mensger et al. 2024[1] (p4 & Table 1, SPIDER structure) |
| Study risk of bias assessment | 11 | Specify the methods used to assess risk of bias in the included studies, including details of the tool(s) used, how many reviewers assessed each study and whether they worked independently, and if applicable, details of automation tools used in the process. | **p3** & Mensger et al. 2024[1] (p6 & Appendix, sec. 7) |
| Effect measures | 12 | Specify for each outcome the effect measure(s) (e.g. risk ratio, mean difference) used in the synthesis or presentation of results. | n.a. |
| Synthesis methods | 13a | Describe the processes used to decide which studies were eligible for each synthesis (e.g. tabulating the study intervention characteristics and comparing against the planned groups for each synthesis (item 5)). | p4–6 of Mensger et al. 2024[1] |
| | 13b | Describe any methods required to prepare the data for presentation or synthesis, such as handling of missing summary statistics, or data conversions. | **p3** & Mensger et al. 2024[1] (p6 & Appendix, sec. 5/6) |
| | 13c | Describe any methods used to tabulate or visually display results of individual studies and syntheses. | n.a. |
| | 13d | Describe any methods used to synthesize results and provide a rationale for the choice(s). If meta-analysis was performed, describe the model(s), method(s) to identify the presence and extent of statistical heterogeneity, and software package(s) used. | n.a. |
| | 13e | Describe any methods used to explore possible causes of heterogeneity among study results (e.g. subgroup analysis, meta-regression). | n.a. |
| | 13f | Describe any sensitivity analyses conducted to assess robustness of the synthesized results. | **p3** & Mensger et al. 2024[1] (p6 & Appendix, sec. 7, CERQual assessment) |
| Reporting bias assessment | 14 | Describe any methods used to assess risk of bias due to missing results in a synthesis (arising from reporting biases). | **p3** & Mensger et al. 2024[1] (p6 & Appendix, sec. 7, CERQual assessment) |
| Certainty assessment | 15 | Describe any methods used to assess certainty (or confidence) in the body of evidence for an outcome. | **p3** & Mensger et al. 2024[1] (p6 & Appendix, sec. 7, CERQual assessment) |

| Topic | No. | Item | Location where item is reported |
|---|---|---|---|
| RESULTS | | | |
| Study selection | 16a | Describe the results of the search and selection process, from the number of records identified in the search to the number of studies included in the review, ideally using a flow diagram. | Mensger et al. 2024[1] (p7, Figure 1, PRISMA flow diagram) |
| | 16b | Cite studies that might appear to meet the inclusion criteria, but which were excluded, and explain why they were excluded. | Mensger et al. 2024[1] (Appendix, sec. 9, list of excluded studies) |
| Study characteristics | 17 | Cite each included study and present its characteristics. | **p4** (Table 1, overview of the included studies) & Mensger et al. 2024[1] (p8, Table 3: Main characteristics of the included 16 studies; p9–13, Table 4: Description of the included articles; Appendix, sec. 8, list of included studies) |
| Risk of bias in studies | 18 | Present assessments of risk of bias for each included study. | **p10-14** (Table 3, Summary of the CERQual assessment for the ten review findings; Appendix, sec. 3/4) & Mensger et al. 2024[1] (p16–21, Table 5: Summary of qualitative findings; Appendix, sec. 11/12) |
| Results of individual studies | 19 | For all outcomes, present, for each study: (a) summary statistics for each group (where appropriate) and (b) an effect estimate and its precision (e.g. confidence/credible interval), ideally using structured tables or plots. | **p4–9** review findings |
| Results of syntheses | 20a | For each synthesis, briefly summarise the characteristics and risk of bias among contributing studies. | **p10-14** (Table 3, Summary of the CERQual assessment for the ten review findings) |
| | 20b | Present results of all statistical syntheses conducted. If meta-analysis was done, present for each the summary estimate and its precision (e.g. confidence/credible interval) and measures of statistical heterogeneity. If comparing groups, describe the direction of the effect. | n.a. (qualitative analysis) |
| | 20c | Present results of all investigations of possible causes of heterogeneity among study results. | n.a. (qualitative analysis) |
| | 20d | Present results of all sensitivity analyses conducted to assess the robustness of the synthesized results. | **p10-14** (Table 3, Summary of the CERQual assessment for the ten review findings; Appendix, sec. 3/4) |
| Reporting biases | 21 | Present assessments of risk of bias due to missing results (arising from reporting biases) for each synthesis assessed. | **p10-14** (Table 3, Summary of the CERQual assessment for the ten review findings; Appendix, sec. 3/4) |
| Certainty of evidence | 22 | Present assessments of certainty (or confidence) in the body of evidence for each outcome assessed. | **p10-14** (Table 3, Summary of the CERQual assessment for the ten review findings; Appendix, sec. 3/4) |
| DISCUSSION | | | |
| Discussion | 23a | Provide a general interpretation of the results in the context of other evidence. | **p15/16** |
| | 23b | Discuss any limitations of the evidence included in the review. | **p16/17** |
| | 23c | Discuss any limitations of the review processes used. | **p16/17** |
| | 23d | Discuss implications of the results for practice, policy, and future research. | **P16/17** |
| OTHER INFORMATION | | | |
| Registration and protocol | 24a | Provide registration information for the review, including register name and registration number, or state that the review was not registered. | **p3** |
| | 24b | Indicate where the review protocol can be accessed, or state that a protocol was not prepared. | **p3** |
| | 24c | Describe and explain any amendments to information provided at registration or in the protocol. | Mensger et al. 2024[1] (Appendix, sec. 1) |
| Support | 25 | Describe sources of financial or non-financial support for the review, and the role of the funders or sponsors in the review. | **p17** |
| Competing interests | 26 | Declare any competing interests of review authors. | **p17** |
| Availability of data, code and other materials | 27 | Report which of the following are publicly available and where they can be found: template data collection forms; data extracted from included studies; data used for all analyses; analytic code; any other materials used in the review. | **p17** |

Page numbers according to the page numbering of the submitted manuscript. [1]Mensger et al. 2024: Mensger C, Jiao Y, Jansky M et al. Voluntarily Stopping Eating and Drinking (VSED): a systematic mixed-methods review focusing on the carers' experiences. Health Policy 2024; 105174.

## 3. Methodological assessments tables (based on Mixed-Methods Appraisal Tool)[3]

The methodological assessment of three publications and the corresponding publications are presented in this appendix. The methodological assessments of all other publications have already been published[1] and are presented in the respective appendix. An overall assessment is not part of the MMAT, but was added here (last column) to facilitate the summary of the appraisal. When assessing methodological limitations in the frame of the CERQual approach, our goal is not to judge whether some absolute standard of methodological quality has been achieved, but rather to indicate concerns where any methodological limitations have been identified as serious enough to lower our confidence in each review finding.[4] The quality of one study can have different impacts dependent on the respective review finding.

Overall assessment criteria for quantitative studies:

- Low quality: with serious concerns about credibility.
- Moderate quality: Some moderate concerns about credibility.
- Good quality: Some minor concerns about credibility.
- High quality: No or only minor concerns about credibility.

Table S2: Methodological assessments table for three quantitative studies

| Author(s), Year | Are there clear research questions? (S1.) | Do the collected data allow to address the RQ? (S2.) | Is the sampling strategy relevant to address the RQ? (4.1.) | Is the sample representative of the target population? (4.2.) | Are the measurements appropriate? (4.3.) | Is the risk of nonresponse bias low? (4.4.) | Is the statistical analysis appropriate to answer the RQ? (4.5.) | Comments | Overall assess-ment |
|---|---|---|---|---|---|---|---|---|---|
| Hagens et al., 2021[5] | Yes.<br><br>1. What is the frequency of VSED<br>2. What are the demographic and medical characteristics of persons who end their lives through VSED | Yes | Yes.<br><br>Sampling strategy is relevant to address the RQs. | Yes, for both RQs.<br><br>Stratified sample of “all deaths in the Netherlands” to obtain target population (“people ending life themselves”/subfraction “by VSED”).<br><br>Description of target population: A stratified sample of death certificates of persons who died between Aug and Dec 2015. Death certificates were stratified based on the likelihood | Mainly.<br><br>Questionnaire not accessible. Information, that questionnaire was largely similar to previous mortality follow-back studies. two questions focusing on patients intentionally ending their | Probably yes, due to high response rate.<br><br>No measures are reported to estimate nonresponse bias (e.g. measures to receive information about reasons of non-participation). No discussion whether | Yes.<br><br>Statistical analysis including frequency statistics fine.<br><br>Limitations discussed well (for example the discrepancy to other studies regarding the absence of psychosocial or existential suffering in the | *Ethical Approval:* No, but NA in the Netherlands as stated by the author.<br><br>*Summary:*<br>Large nationwide sample, which is representative of all deaths in the Netherlands in 2015. Study with a high response rate and thorough statistical analysis. All RQs were answered in a valid way.<br><br>*Limitation:*<br>Very small subsample (N = 25) of VSED cases. Due to representative sample, this | Good – high quality |

| Author(s), Year | Are there clear research questions? (S1.) | Do the collected data allow to address the RQ? (S2.) | Is the sampling strategy relevant to address the RQ? (4.1.) | Is the sample representative of the target population? (4.2.) | Are the measurements appropriate? (4.3.) | Is the risk of nonresponse bias low? (4.4.) | Is the statistical analysis appropriate to answer the RQ? (4.5.) | Comments | Overall assess-ment |
|---|---|---|---|---|---|---|---|---|---|
| | | | | that the death had been preceded by an end-of-life decision. Further details about stratification procedure is missing. However, this could be obtained from previous follow-back studies from authors. Additionally, no size was given for the stratum suicide. Questionnaires were sent to physicians attending stratified deaths (n=9351)<br><br>Clear description of sample population: Physicians attending deaths which were suicide or where the physician had indications that the patient intentionally ended one's own life. Clear description of eligible sample (N = 521; subsample VSED N = 25). Excluded respondents were specified by number & reasons (N = 8).<br><br>*Sample (2. RQ):* Physicians attending deaths which were suicide or where the physician had indications that the | own life were cited.<br><br>Questionnaire from previous follow-back studies were validated (information about that in previous follow-back studies of authors). However, it was not fully made **clear** which questions were already published and which were new (e.g. questions about medical conditions & characteristics of the patient) and if they were validated or tested. | respondents & non-respondents are equal or not.<br><br>Response rate: 78%<br><br>Respondents could be more interested in the topic of end-of-life decisions if they have experience in that *(not discussed in the paper; relevant for 1. RQ).* | group of people who had ended life by VSED, which was identified in the other studies as playing a significant role in the patients' motives). | could be less relevant. However, as random variations in the measures would have a greater effect compared with larger sample sizes, this was judged as minor concern leading to rating good-high instead to high quality. | |

| Author(s), Year | Are there clear research questions? (S1.) | Do the collected data allow to address the RQ? (S2.) | Is the sampling strategy relevant to address the RQ? (4.1.) | Is the sample representative of the target population? (4.2.) | Are the measurements appropriate? (4.3.) | Is the risk of nonresponse bias low? (4.4.) | Is the statistical analysis appropriate to answer the RQ? (4.5.) | Comments | Overall assess-ment |
|---|---|---|---|---|---|---|---|---|---|
| | | | | patient intentionally ended one's own life. | | | | | |
| Stängle et al. 2019b[6] *see also Stängle et al. 2021b*[7] | Yes. See *Stängle et al. 2021b*<br><br>The aim was to describe people who have chosen VSED and their course of dying *(focus on oncological VSED patients)* | Yes. See *Stängle et al. 2021b* | Yes. See *Stängle et al. 2021b* | Partly.<br>*See also Stängle et al. 2021b*<br><br>Subsample of nationwide survey: HCPs (with VSED experience: N = 627) from the settings: primary care, outpatient care, long-term care.<br><br>N = 254<br>(subsample of oncological VSED patients) | Yes.<br><br>The questionnaire is accessible and the questions correspond to the statements in the article.<br><br>Further details, see *Stängle et al. 2021b* | No. See *Stängle et al. 2021b*<br>Altogether, the risk of nonresponse bias cannot be assessed as "low". | Yes, mainly.<br><br>Descriptive statistics in principle fine. Frequency measures in % are reported. However further details about statistical analysis is missing (e.g. missing values; mean; standard deviation, data about the comparison with other patients with other diseases) | *Ethical Approval:* See *Stängle et al. 2021b*<br><br>*Summary:* No research article (e.g. no division into methods, results and discussion section), but short article presenting an extract of results from the nationwide survey (*see Stängle et al.*<br>2021b) on the subsample of oncology patients.<br>*Limitations:*<br>Still questions about the representativeness of the sample. It is unclear if results can be transferred to other settings (hospice, hospital). Because it is not a research paper and does not provide all relevant information and tests, the quality was downgraded to moderate. | Moderate quality |
| Stängle et al. 2019c[8] *see also Stängle et al. 2021b*[7] | Yes. See *Stängle et al. 2021b*<br><br>The aim was to describe people who have chosen VSED and to make | Yes. See *Stängle et al. 2021b* | Yes. See *Stängle et al. 2021b* | Partly.<br>*See also Stängle et al. 2021b*<br><br>Subsample of nationwide survey: HCPs (with VSED experience: N = 627) from the settings: primary care, | Unclear.<br><br>The questionnaire is accessible. No information found in the questionnaire which VSED | No. See *Stängle et al. 2021b*<br>Altogether, the risk of nonresponse bias cannot be assessed as "low". | Yes.<br><br>Descriptive statistics fine and detailed presentation of data in tables, broken down for the individual | *Ethical Approval:* See *Stängle et al. 2021b*<br><br>*Limitation:*<br>Validity of quantitative variables is unclear and elicits serious concern. Because according to the article, the study participants should describe | Low quality |

| Author(s), Year | Are there clear research questions? (S1.) | Do the collected data allow to address the RQ? (S2.) | Is the sampling strategy relevant to address the RQ? (4.1.) | Is the sample representative of the target population? (4.2.) | Are the measurements appropriate? (4.3.) | Is the risk of nonresponse bias low? (4.4.) | Is the statistical analysis appropriate to answer the RQ? (4.5.) | Comments | Overall assess-ment |
|---|---|---|---|---|---|---|---|---|---|
| | statements about the course of the accompaniment. | | | outpatient care, long-term care.<br><br>N = 270 family physicians;<br>N = 166 nurses outpatient care;<br>N = 191 nurses long-term care | case (last case or other) should be reported. Discrepancy with the published article where it is described that study participants should describe their last or most memorable case which leads to a strong risk of recall bias and compromises quantitative statements.<br><br>Further details, see *Stängle et al. 2021b* | | diseases and morbidity pattern. | their last or memorably VSED case which leads to a strong risk of recall bias regarding quantitative statements about the accompanied VSED case, especially in regards of the characteristic of the patients. As this is the main topic of the article the quality was downgraded to low quality.<br>In addition, the RQ was related to people who have chosen VSED in general and was not limited to a specific setting. Patient characteristics might be different if additional VSED settings such as hospices and hospitals had been included *(as discussed in the article*). This is relevant to the frequency distribution in relation to the different diseases and morbidity patterns. Therefore, confidence in the measures and their generalization is not without concern. | |

HCP: Healthcare professionals; VSED: Voluntary Stopping of Eating and Drinking. RQ: Research question. N: sample size.

## 4. Evidence Profile Table of the CERQual assessment[9]

Table S3: Evidence Profile Table of the CERQual assessment

| # | Summarized review finding | Methodological limitations | Coherence | Adequacy | Relevance | GRADE-CERQual assessment of confidence | References[5–8,10–27] |
|---|---|---|---|---|---|---|---|
| **A: MOTIVES AND DECISION MAKING** | | | | | | | |
| 1 | ***The motives – high symptom burden and suffering**:* Individuals opting for VSED typically have a high symptom burden and an accumulation of health problems, which finally result in a reduced quality of life and deterioration of health status. Psychosocial, spiritual or existential factors like “emerging life fatigue” or “meaninglessness of life” also play a role in the decision-making process for VSED. Avoidance of suffering can also refer to the future as described for early dementia. | Minor concerns<br><br>**Explanation:** Minor concerns regarding methodological limitations, as one study (Stängle et al. 2019c) was rated as "low quality" because the sampling strategy for VSED cases has a high risk of recall bias and the quantification of the statements in this study is not without concern.<br>As the aspects of the review finding supported by this study were also supported by the other studies, the concerns regarding methodological limitations for the overall review finding were rated as minor. | Minor concerns<br><br>**Explanation:** Minor concerns regarding coherence. Three studies (Stängle et al. 2019c; Bolt et al. 2015, Fringer et al. 2020) are reporting that "psychosocial, spiritual or existential factors" play a role in VSED decision making. But one study (Hagens et al. 2021) is reporting contradictory data (that "only 3% of VSED people had psychosocial or existential problems (N=25)). As this is only one study with a small sample size and the other three studies are very coherent, the concerns were jugded as minor. The finding is more descriptive. | No/Very minor concerns<br><br>**Explanation:** This review finding is based on data from 10 of the 22 included publications, which is considered as solid quantity. Additionally, there is a balanced mix of quantitative and qulitative data supporting the main aspects of the review finding. Therefore no concerns. | Serious concerns<br><br>**Explanation:** Serious concerns regarding relevance mainly because of indirect evidence which is considered especially important here, as the finding itselfs is a statement made from the perspective of VSED persons. But no data of affected people was available at the timepoint of data analysis . In the quantitative studies the experiences of the attending physicians were captured and in qualitative studies the experience of caring relatives and healthcare professionals. Additionall concerns because the setting of the RQ is „global“, but only some countries (Switzerland, Germany, Netherlands, US) are contributing to the finding (partial relevance). | Low confidence<br><br>**Explanation:** Minor concerns regarding methodological limitations, minor concerns regarding coherence, no/very minor concerns regarding adequacy, and serious concerns regarding relevance because of indirect evidence (finding about motives of VSED persons, but data from attending physicians, nurses and relatives) and partial relevance (global research question, but review finding based only on data from a few countries). | Bolt et al. 2015;<br>Eppel-Meichlinger et al. 2021;<br>Fringer et al. 2020;<br>Hagens et al. 2021;<br>Lowers 2020;<br>Lowers et al. 2021;<br>Saladin et al. 2018;<br>Stängle et al. 2019b;<br>Stängle et al. 2019c;<br>Starke 2020 |

| # | Summarized review finding | Methodological limitations | Coherence | Adequacy | Relevance | GRADE-CERQual assessment of confidence | References[5–8,10–27] |
|---|---|---|---|---|---|---|---|
| 2 | ***The circumstances of the VSED decision:*** Persons who wished to die by VSED often had a terminal illness. Most patients had severe disease (76%) and a life expectancy of less than one year (74%). The transition from symptoms and disease progression to the final decision of VSED can be fluid, with conscious support for the body's declining constitution. However, patients also experience deterioration in their health without being in a terminal stage, and one-third of patients did not suffer from a severe illness (29%). Not all VSED patients (90%) were assessed as competent to opt for VSED. Ethical case consultation was conducted in 27% of cases, and a psychiatric consultation in less than 10% of VSED cases. | Minor concerns<br><br>**Explanation:** Minor concerns regarding methodological limitations, as one study (Stängle et al. 2019c) was rated as "low quality" because the sampling strategy for VSED cases has a high risk of recall bias and the quantification of the statements in this study is not without concern.<br>As this study only supports an individual aspect of several individual aspects, the concerns regarding methodological limitations for the overall review finding were rated as minor. | No/Very minor concerns<br><br>**Explanation:** Finding is very descriptive. Finding consists of a couple of individual aspects, but all in all, single aspects are coherent among each other. No contradictory and no ambiguous data. | Moderate concerns<br><br>**Explanation:** Moderate concerns regarding adequacy because most individual aspects of the review finding are based on only one study. Some quantitative aspects were based on qualitative studies only, which should be verified by further quantitative research. | Moderate concerns<br><br>**Explanation:** Moderate concerns regarding relevance because: The setting of the research question is „global“ and only a few countries (Switzerland, Germany, Netherlands, US) are contributing to the finding (partial relevance), especially as the finding consists of several individual aspects that are usually only supported by one study from one country. | Moderate confidence<br><br>**Explanation:** Minor concerns regarding methodological limitations, no/very minor concerns regarding coherence, moderate concerns regarding adequacy (missing data quantity for the individual aspects of the review finding), and moderate concerns regarding relevance (global research question, but review finding based only on data from a few countries). | Bolt et al. 2015;<br>Fringer et al. 2020;<br>Hoekstra et al. 2015;<br>Hoekstra 2020;<br>Lowers 2020;<br>Stängle et al. 2019b;<br>Stängle et al. 2019c;<br>Starke 2020 |
| 3 | ***Self-determination and autonomy:*** Frequently reported motives for VSED are often associated with autonomy and independence and the fear of losing them during the disease (57%–60% of the motives). Retaining control over the | Minor concerns<br><br>**Explanation:** Minor concerns regarding methodological limitations, as one study (Stängle et al. 2019c) was rated as "low quality" | No/Very minor concerns<br><br>**Explanation:** The review finding is more descriptive than interpretative and covers one main aspect: self- | No/Very minor concerns<br><br>**Explanation:** This review finding is based on data from 11 of the 22 included publications, | Moderate concerns<br><br>**Explanation:** Moderate concerns regarding relevance, because: The setting of the review question is "global" and only some countries (Switzerland, Germany, Netherlands, USA) | Moderate confidence<br><br>**Explanation:** Minor concerns regarding methodological limitations, no/very minor concerns regarding coherence, no/very minor | Bolt et al. 2015;<br>Eppel-Meichlinger et al. 2021;<br>Fringer et al. 2020;<br>Gerson et al. 2020;<br>Lowers 2020;<br>Lowers et al. 2021;<br>Malpas & Mitchell 2017; |

| # | Summarized review finding | Methodological limitations | Coherence | Adequacy | Relevance | GRADE-CERQual assessment of confidence | References[5–8,10–27] |
|---|---|---|---|---|---|---|---|
| | dying process is desired; the last resort is expressing one's will. Accordingly, persons who have chosen VSED are described as strong-minded, a characteristic that the course of VSED itself requires. | because the sampling strategy for VSED cases has a high risk of recall bias and the quantification of the statements in this study is not without concern.<br>As the aspect of the review finding supported by this study were also supported by other studies, the concerns regarding methodological limitations for the overall review finding were rated as minor. | determination and autonomy. Very coherent data as all studies support the direction of the message, namly that self-determination and autonomy is an important aspect of decision-making. | which is considered as „solid quantity". Additionally, there is a balanced mix of quantitative and qualitative data supporting the review finding. Therefore no concerns. | contribute to the review finding (partial relevance), mainly data from Switzerland and the Netherlands. In addition, relevant data cames from a study (Fringer et al. 2020) with the main topic "food refusal" and only partially about VSED (indirect relevance). However, as this study supports the main message and due to the amount of other data, this was considered as minor. | concerns regarding adequacy, and moderate concerns regarding relevance (global research question, but review finding based only on data from a few countries). | Saladin et al. 2018;<br>Stängle et al. 2019b;<br>Stängle et al. 2019c;<br>Starke 2020 |
| 4 | ***VSED as the "best available option" or "better than other options":*** The option of ending life through VSED was often pragmatically regarded as the "best option available" or "better than other end-of-life options." Compared to the decision to commit suicide, VSED was preferred. The same was true if it was the only option when Physician- or Medical Assisted Suicide was not available (e.g., because of jurisdiction, no terminal condition, costs). Additionally, | No/Very minor concerns<br><br>**Explanation:** No methodological concerns identified which could have an impact on the review finding. | No/Very minor concerns<br><br>**Explanation:** The review finding is more descriptive. The finding consists of two main aspects (best available option & better than other options) and is underpinned by individual aspects that support the direction of these main aspects. Data very coherent. No | Minor concerns<br><br>**Explanation:** Minor concerns regarding adequacy because: Most studies supporting the finding are qualitative studies. The question "Why do people choose VSED compared to other end-of-life options such as MAiD or PAS" could have | Moderate concerns<br><br>**Explanation:** Moderate concerns regarding relevance because: The setting of the review question is "global" and only a few countries (Switzerland, Netherlands, USA) contribute to the review finding (partial relevance). Nevertheless, it seems likely that the content of the review result (best available option) is independent of the context and would not change | Moderate confidence<br><br>**Explanation:** No/very minor concerns regarding methodological limitations, no/very minor concerns regarding coherence, minor concerns regarding adequacy (could be more quantitative data), and moderate concerns regarding relevance (global research question, | Bolt et al. 2015;<br>Gerson 2018;<br>Gerson et al. 2020;<br>Hagens et al. 2021;<br>Lowers 2020;<br>Lowers et al. 2021;<br>Saladin et al. 2018;<br>Stängle & Fringer 2021a |

| # | Summarized review finding | Methodological limitations | Coherence | Adequacy | Relevance | GRADE-CERQual assessment of confidence | References[5–8,10–27] |
|---|---|---|---|---|---|---|---|
| | VSED was also chosen to experience the dying process consciously. | | contradictory and no ambiguous data. | been further supported by quantitative studies. | significantly if data from more countries were included. | but review finding based only on data from a few countries). | |
| **B: ATTITUDES TO VSED FROM HEALTHCARE PROFESSIONALS AND RELATIVES** | | | | | | | |
| 5 | ***Attitudes of healthcare professionals (HCPs):*** HCPs seems to be open to VSED. They (>90%) largely accept the person's decision for VSED and perceive it as understandable. Although they also report that "food refusal" can be a challenging topic. Most HCPs consider dying by VSED as compatible with their worldview or religion (86%) and as compatible with the culture of their facility or their professional ethics (69%–70%). Despite these positive attitudes, physicians (34%–61%) and nurses (33%–48%) are more reluctant to recommend VSED. Although the majority (77%) of HCPs believe it is important to judge the patients' mental capacity when they opt for VSED, a fifth (22%) take a neutral position or believe this is unnecessary. This judgment was significantly more critical for HCPs without VSED experience than those with experience. Furthermore, attitudes may differ across | No/Very minor concerns<br><br>**Explanation:** No methodological concerns identified which could have an impact on the review finding. | Minor concerns<br><br>**Explanation:** The review finding is very descriptive and covers a couple of individual aspects. Minor concerns regarding coherence because: There is conflicting information between the Stängle studies (Switzerland) and the study (Bolt et al. 2015) from the Netherlands regarding the recommendation of VSED to patients. These discrepancies may be due to a lack of clarity and consistency between the studies as to what situation the patient was in, whether the patient had previously requested PAS or whether the patient had generally | Moderate concerns<br><br>**Explanation:** Moderate concerns regarding adequacy because: 10 out of 22 studies contribute to the review finding. Most of them are quantitative studies, which raises some concerns about the richness of the data. In addition, most aspects of the review finding are based on just one study from Switzerland (in some cases on several publications, but from one nationwide study). However, the aspect of "high acceptance of VSED in general" | Moderate concerns<br><br>**Explanation:** Moderate concerns regarding relevance, because: The setting of the review question is "global" and only a few countries (Switzerland, Germany, Netherlands, USA) contribute to the review finding (partial relevance). Some aspects are mainly based on one nationwide study from Switzerland. Furthermore, given the cultural differences between countries and societies in relation to end-of-life options, it is not unlikely that there are different attitudes that have not been captured by the underlying and available data. Given that VSED (and end-of-life options in general) is a controversial topic, this raises concerns. However, as quantitative studies from three countries (Switzerland, Germany, Netherlands) clearly support a main aspect of the review finding („high acceptance in general"), the overall concerns regarding | Low confidence<br><br>**Explanation:** No/very minor concerns regarding methodological limitations, minor concerns regarding coherence, moderate concerns regarding adequacy (mainly quantitative data; some aspects based largely on one nationwide study), and moderate concerns regarding relevance (it is not unlikely that the results regarding attitudes to this controversial topic will change if studies from other and more critical countries are included). | Bolt et al. 2015; Fringer et al. 2020; Gerson 2018; Hoekstra et al. 2015; Hoekstra 2020; Stängle et al. 2020a; Stängle et al. 2020b; Stängle & Fringer 2021a; Stängle et al. 2021b; Stängle et al. 2021c |

| # | Summarized review finding | Methodological limitations | Coherence | Adequacy | Relevance | GRADE-CERQual assessment of confidence | References[5–8,10–27] |
|---|---|---|---|---|---|---|---|
| | language and cultural regions. | | expressed a wish to die. As this is only a single aspect of the review finding, it is considered a minor concern. | has a high approval rate in all studies (nationwide studies from Stängle; Hoekstra et al. 2015; Bolt et al. 2015). Therefore, moderate (and not serious) concerns regarding the overall review finding. | relevance were considered moderate and not serious. | | |
| 6 | ***Attitudes towards VSED as natural dying or suicide:*** Attitudes on VSED varies. Most healthcare professionals (63%) view VSED as a natural death, with more nurses (67%) than physicians (59%) consider it that way. In addition, VSED is equated with "letting die" by a proportion of professionals (27%), and a minority regard it as "something else" (about 5%). A minority (about 5%) see VSED as (assisted) suicide. Most VSED cases (81%–99%) are documented as "natural death" on the death certificate. In addition to healthcare professionals, it was also reported that relatives did not classify their loved one's VSED as an act of self-killing. The | Minor concerns<br><br>**Explanation:** Minor concerns regarding methodological limitations, as one study (Stängle et al. 2019c) was rated as "low quality" because the sampling strategy for VSED cases has a high risk of recall bias and the quantification of the statements in this study is not without concern.<br>As this study only supports an individual aspect of several individual aspects, the concerns regarding methodological | Moderate concerns<br><br>**Explanation:** The review finding is more descriptive, but also have interpretative aspects (decision to consider the range of attitudes as the main statement). Moderate concerns because: There is conflicting data between Hoekstra et al. 2015 and the nationwide study from Stängle et al. on the question of whether the classification of VSED as "natural dying" or "suicide" depends on the | Moderate concerns<br><br>**Explanation:** This review finding is based on data from 12 of the 22 included publications, which is considered as „solid quantity" in principle, but there are moderate concerns regarding adequacy because: The main message of the review finding is mainly based on a nationwide study from Switzerland. However, | Moderate concerns<br><br>**Explanation:** Moderate concerns regarding relevance because: The setting of the review question is "global" and only a few countries (Switzerland, Germany, Netherlands, USA) contribute to the review finding (partial relevance), mainly data from Switzerland. As the main message of the review finding is the range of attitudes, this is considered as a moderate and not as a serious concern. | Very low confidence<br><br>**Explanation:** Minor concerns regarding methodological limitations, moderate concerns regarding coherence (contradictory data and alternative explanations with two individual aspects), moderate concerns regarding adequacy (should be more quantity and richness of the data), and moderate concerns regarding relevance (it is not unlikely that the results regarding attitudes to this controversial topic will change if studies from | Fringer et al. 2020;<br>Gerson et al. 2020;<br>Hagens et al. 2021;<br>Hoekstra et al. 2015;<br>Saladin et al. 2018;<br>Stängle et al. 2019c;<br>Stängle et al. 2020a;<br>Stängle et al. 2020b;<br>Stängle & Fringer 2021a;<br>Stängle et al. 2021b;<br>Stängle et al. 2021c;<br>Starke 2020 |

| # | Summarized review finding | Methodological limitations | Coherence | Adequacy | Relevance | GRADE-CERQual assessment of confidence | References[5–8,10–27] |
|---|---|---|---|---|---|---|---|
| | assessment of VSED may also depend on the specific case and circumstances (e.g., age, disease, and life expectancy of the VSED person). | limitations for the overall review finding were rated as minor. | individual case. Furthermore, there could also be other reasons for the fact that most VSEDs are documented as natural deaths on the death certificate, e.g. medico-legal reasons. Overall, this led to moderate concerns. | individual aspects are supported by other individual studies (quantitative and qualitative). Nevertheless, more quantitative and qualitative data to support this finding and all its individual aspects would increase its reliability. | | other countries are included). | |
| 7 | ***Acceptance and its influencing factors:*** The acceptance of a VSED decision by healthcare professionals (HCPs) and relatives depends on several factors:<br>(a) **Patient characteristics*:*** Acceptance is primarily related to the person's health status. For example, in the situation of a terminally ill patient, VSED is widely accepted. In contrast, in the case of a person in good health or without visible disease, it is less accepted. Acceptance also appears to increase with the age of the person concerned.<br>(b) **Assessment of whether VSED is suicide:** The assessment of the situation | No/Very minor concerns<br><br>**Explanation:** No methodological concerns identified which could have an impact on the review finding. | No/Very minor concerns<br><br>**Explanation:** The review finding is more descriptive but also have interpretative aspects, particularly the summary that acceptability depends on four factors. There are no concers about coherence as the range of experiences and attitudes are captured in the review finding (see full review finding in text). | Moderate concerns<br><br>**Explanation:** Moderate concerns regarding adequacy because: This review finding is based on data from 12 of the 22 included publications with a mix of quantitative and qualitative studies, which is generally considered a "solid quantity". However, there are moderate concerns about | Moderate concerns<br><br>**Explanation:** Moderate concerns regarding relevance because: The setting of the review question is „global" and only a few countries (Switzerland, Germany, US, New Zealand) contribute to the finding (partial relevance). As the review finding consists of different aspects, the number of countries contributing to these aspects is even smaller, which raises concerns. | Low confidence<br><br>**Explanation:** No/very minor concerns regarding methodological limitations, no/very minor concerns regarding coherence, moderate concerns regarding adequacy, and moderate concerns regarding relevance (global research question, but the review finding is only based on data from a few countries, especially when looking at the individual aspects of the review findings). | Eppel-Meichlinger et al. 2021;<br>Fringer et al. 2020;<br>Gerson 2018;<br>Gerson et al. 2020;<br>Hoekstra et al. 2015;<br>Lowers 2020;<br>Lowers et al. 2021;<br>Malpas & Mitchell 2017;<br>Saladin et al. 2018;<br>Stängle et al. 2020a;<br>Stängle & Fringer 2021a;<br>Stängle et al. 2021b |

| # | Summarized review finding | Methodological limitations | Coherence | Adequacy | Relevance | GRADE-CERQual assessment of confidence | References[5–8,10–27] |
|---|---|---|---|---|---|---|---|
| | as suicide by HCPs, relatives, and facilities may lead to their rejection of VSED, whereas the definition of natural dying generally leads to more acceptance.<br>(c) **Circumstances surrounding the decision:** Traceability of motives and knowing the patient and their situation well were critical to promote acceptance. Furthermore, reasons related to self-determination and independence were generally well accepted by HCPs and relatives.<br>(d) **Personal characteristics:** Sometimes, personal attitudes, cultural background, or religious beliefs do not allow acceptance of VSED. | | | adequacy because: The review finding consists of four different individual aspects and more quantitative and qualitative data on these respective individual aspects should support the reliability of the statements. | | | |
| **C: ADVOCACY DURING ACCOMPANIMENT AND RELATIVE'S GRIEF** | | | | | | | |
| 8 | ***Advocacy by healthcare professionals (HCPs):*** Most VSED cases (76%) are accompanied by HCPs, and their willingness to accompany a person during a VSED is high (>90%). Once committed to the patient's wish, they can evolve into an advocate for the VSED person. When caregiving has been ongoing for years, the decision to provide accompaniment is evident to | Minor concerns<br><br>**Explanation:** Minor concerns regarding methodological limitations, as one study (Stängle et al. 2019c) was rated as "low quality" because the sampling strategy for VSED cases has a high risk of recall bias and the | No/Very minor concerns<br><br>**Explanation:** The review finding is mainly descriptive and covers one main aspect (advocacy & commitment by HCPs). Very coherent data as all studies support the | Minor concerns<br><br>**Explanation:** Minor concerns regarding adequacy because: This review finding is based on data from 9 of the 22 included publications and mainly from 4 qualitative studies, | Moderate concerns<br><br>**Explanation:** Moderate concerns regarding relevance, because: The setting of the review question is "global" and only two countries (Switzerland, USA) contribute to the review finding (partial relevance). As the content of the review finding deals with caring, professional self-conception and solidarity, it is | Moderate confidence<br><br>**Explanation:** Minor concerns regarding methodological limitations, no/very minor concerns regarding coherence, minor concerns regarding adequacy, and moderate concerns regarding relevance (global research question, | Gerson et al. 2020;<br>Lowers 2020;<br>Saladin et al. 2018;<br>Stängle et al. 2019c;<br>Stängle et al. 2020a;<br>Stängle et al. 2020b;<br>Stängle & Fringer 2021a;<br>Stängle et al. 2021b; |

| # | Summarized review finding | Methodological limitations | Coherence | Adequacy | Relevance | GRADE-CERQual assessment of confidence | References[5–8,10–27] |
|---|---|---|---|---|---|---|---|
| | them. They are committed to the patient's needs and wishes and do everything they can to support the patients. Advocacy may be to the point where HCPs act independently of the team or feel compelled to act contrary to the facility's decision (in case of interdiction of VSED accompaniment). The patient's "happiness" and trust make care and engagement even more accessible, as they reported. | quantification of the statements in this study is not without concern.<br>As this study only supports an individual aspect of several individual aspects, the concerns regarding methodological limitations for the overall review finding were rated as minor. | direction of the message. | which raises some concerns. However, these concerns are considered minor as the data were considered quite rich for the main message of the review finding. Furthermore, the lack of quantitative data does not raise too much concern as the content of the review finding is best captured by qualitative research. | suspected that it differes not so much between countries. | but review finding based only on data from two countries). As the content of the review finding deals with caring, professional self-conception and human solidarity and less about attitudes, the differences between the countries were judged not to be such great. | Stängle et al. 2021c |
| 9 | ***Advocacy by relatives:*** Just over half (58%) of VSED cases are accompanied by relatives. They often play an active role, doing whatever is necessary for the patient: speaking for and on behalf of the patient, making logistical arrangements, organizing the farewells to family and friends, protecting the patient from exposure to tempting food and drink and the forced administration of the same. They discuss and debate with professionals or other family members to ensure their loved one's will is respected. | Minor concerns<br><br>**Explanation:** Minor concerns regarding methodological limitations, as one study (Stängle et al. 2019c) was rated as "low quality" because the sampling strategy for VSED cases has a high risk of recall bias and the quantification of the statements in this study is not without concern. | No/Very minor concerns<br><br>**Explanation:** The review finding is mainly descriptive and covers one main aspect (advocacy & engagement by relatives) consisting of some subaspects. Very coherent data as all studies support the direction of the message. | Moderate concerns<br><br>**Explanation:** Moderate concerns regarding adequacy because: This review finding is based on data from 5 of the 22 included publications and mainly from only two qualitative studies, which raises concerns. | Moderate concerns<br><br>**Explanation:** Moderate concerns regarding relevance because: The setting of the review question is "global" and only two countries (Switzerland, USA) contribute to the review finding (partial relevance). As the content of the review finding deals with caring, human solidarity and family cohesion, it is not considered to vary as much between countries as, for example, attitudes, which are considered to be more | Moderate confidence<br><br>**Explanation:** Minor concerns regarding methodological limitations, no/very minor concerns regarding coherence, moderate concerns regarding adequacy (individual aspects should be supported by further research), and moderate concerns regarding relevance (global research question, but review finding | Eppel-Meichlinger et al. 2021;<br>Lowers 2020;<br>Lowers et al. 2021;<br>Saladin et al. 2018;<br>Stängle et al. 2019c |

| # | Summarized review finding | Methodological limitations | Coherence | Adequacy | Relevance | GRADE-CERQual assessment of confidence | References[5–8,10–27] |
|---|---|---|---|---|---|---|---|
| | They do all this despite their own grief and resistance, such as legal or moral accusations. These obstacles have even led them to become advocates. With diminishing capacity and unconsciousness, the role of advocacy is even more important to ensure a successful VSED. | As this study only supports an individual aspect of several individual aspects, the concerns regarding methodological limitations for the overall review finding were rated as minor. | | These concerns are considered moderate and not serious as the data were considered quite rich for the main message of the review finding. However, the individual aspects could be supported by further research. Furthermore, the lack of quantitative data does not raise too much concern as the content of the review finding is best captured by qualitative research. | dependent on cultural and religious background and education. | based only on data from two countries). As the content is more about human solidarity and family cohesion and less about attitudes, the differences between the countries were judged not to be such great. | |
| 10 | ***The grieving process after VSED accompaniment:*** In the case of a terminal illness, grief after a VSED is not perceived as more complicated than without VSED. Compared to suicide, it is rated as “better” because of the opportunity to say goodbye and seek professional support. Relatives provide tireless support, which leads to excessive exhaustion. During | No/Very minor concerns<br><br>**Explanation:** No methodological concerns identified which could have an impact on the review finding. | No/Very minor concerns<br><br>**Explanation:** The review finding is more descriptive. The finding consists of several aspects. As the whole range of experiences are captured in the reveiw finding, no concerns about coherence. | Moderate concerns<br><br>**Explanation:** Moderate concerns regarding adequacy because: This review finding is based on data from 3 of the 22 included publications, | Moderate concerns<br><br>**Explanation:** Moderate concerns regarding relevance because: The setting of the review question is "global" and only a few countries (Switzerland, Germany, New Zealand) contribute to the review finding (partial relevance). As the content of the review finding deals with a fundamental human topic | Moderate confidence<br><br>**Explanation:** No/very minor concerns regarding methodological limitations, no/very minor concerns regarding coherence, moderate concerns regarding adequacy (review content should be supported by further research), | Eppel-Meichlinger et al. 2021;<br>Malpas & Mitchell 2017;<br>Starke 2020 |

| # | Summarized review finding | Methodological limitations | Coherence | Adequacy | Relevance | GRADE-CERQual assessment of confidence | References[5–8,10–27] |
|---|---|---|---|---|---|---|---|
| | the grieving phase, they “recharge their batteries” and reflect on what has happened. Relatives can feel fulfilled, satisfied and grateful depending on how they assess their accompaniment and the dying situation. However, they may experience a more difficult grief, for example, when the VSED person had suffered, when the death was agonizing, when they have experienced a situation not manageable due to lack of support, or when they are struggling with their support. If the wish to hasten death was not understandable, or if they reject VSED as suicide, they have to fight with feelings of guilt and self-blame. These negative experiences and assessments can lead to traumatic memories and distress. | | | mainly from two qualitative studies, which raises concerns. These concerns are considered moderate and not serious as the data were considered quite rich for the main message ("grief varies and is dependent on own assessment & situation"). However, the individual aspects could be supported by further research. Furthermore, the lack of quantitative data does not raise too much concern as the content of the review finding is best captured by qualitative research. | and need, it is not considered to vary as much between countries as, for example, attitudes, which are considered to be more dependent on cultural and religious background and education. | and moderate concerns regarding relevance (global research question, but review finding based only on data from two countries). As the content is more about human solidarity and family cohesion and less about attitudes, the differences between the countries were judged not to be such great. | |

VSED, voluntary stopping of eating and drinking; HCPs, healthcare professionals

## 5. References


1. [48] Mensger C, Jiao Y, Jansky M et al. Voluntarily Stopping Eating and Drinking (VSED): a systematic mixed-methods review focusing on the carers' experiences. Health Policy 2024; 105174.
2. [49] Page MJ, McKenzie JE, Bossuyt PM, et al. The PRISMA 2020 statement: an updated guideline for reporting systematic reviews. *BMJ 2021*; 372:n71.
3. [59] Hong QN, Pluye P, Fàbregues S, et al. Mixed Methods Appraisal Tool (MMAT), version 2018. User guide. Registration of Copyright (#1148552), Canadian Intellectual Property Office, Industry Canada. Available at: http://mixedmethodsappraisaltoolpublic.pbworks.com/w/file/fetch/146002140/MMAT_2018_criteria-manual_2018-08-08c.pdf (accessed October 31 2024).
4. [54] Munthe-Kaas H, Bohren MA, Glenton C, et al. Applying GRADE-CERQual to qualitative evidence synthesis findings – paper 3: how to assess methodological limitations. *Implementation Sci* 2018; 13(Suppl 1): 25–32.
5. [71] Hagens M, Pasman HRW, van der Heide A, et al. Intentionally ending one's own life in the presence or absence of a medical condition: A nationwide mortality follow-back study. *SSM Popul Health* 2021; 15: 100871.
6. [72] Stängle S, Benedetti FD and Fringer A. "Sterbefasten": Freiwilliger Verzicht auf Nahrung und Flüssigkeit in der Onkologie. ["Fasting to Death": Voluntary stopping of eating and drinking in Oncology]. *Onkologiepflege* 2019b; 17–20.
7. [17] Stängle S, Büche D, Häuptle C, et al. Experiences, Personal Attitudes, and Professional Stances of Swiss Health Care Professionals Toward Voluntary Stopping of Eating and Drinking to Hasten Death: A Cross-Sectional Study. *J Pain Symptom Manage* 2021b; 61: 270–78.
8. [11] Stängle S, Schnepp W, Büche D, et al. Pflegewissenschaftliche Erkenntnisse über die Betroffenen, den Verlauf und der Begleitung beim freiwilligen Verzicht auf Nahrung und Flüssigkeit aus einer standardisierten schweizerischen Gesundheitsbefragung [Nursing findings on the affected persons, the course and the accompaniment of voluntary stopping of eating and drinking from a standardized Swiss health survey]. *ZfmE* 2019c; 65: 237–248.
9. [53] Lewin S, Booth A, Glenton C, et al. Applying GRADE-CERQual to qualitative evidence synthesis findings: introduction to the series. *Implementation Sci* 2018; 13(Suppl 1): 1–10.
10. [64] Eppel-Meichlinger J, Stängle S, Mayer H, et al. Family caregivers' advocacy in voluntary stopping of eating and drinking: A holistic multiple case study. *Nurs Open* 2021; 00: 1–13.
11. [63] Lowers J, Hughes S and Preston N. Experience of Caregivers Supporting a Patient through Voluntarily Stopping Eating and Drinking. *J Palliat Med* 2021; 24: 376–81.
12. [60] Lowers J. Experiences of caregivers who support a patient who elects voluntarily stopping eating and drinking (VSED) to hasten death. Dissertation 2020. Lancaster University (United Kingdom).
13. [73] Stängle S and Fringer A. Perspectives of people accompanying a person during voluntary stopping eating and drinking: a convergent mixed methods study. *Ann Palliat Med* 2021a; 10: 1994–2007.
14. [66] Fringer A, Stängle S, Büche D, et al. The associations of palliative care experts regarding food refusal: A cross-sectional study with an open question evaluated by triangulation analysis. *PLoS One* 2020; 15: e0231312.
15. [67] Starke P. Freiwilliger Verzicht auf Essen und Trinken – Zur ethischen Lagebestimmung eines ambivalenten Begriffs. [Voluntary stopping of eating and drinking – On tackling its ethical ambivalence]. *Ethik Med* 2020; 32: 171–187.
16. [18] Stängle S, Schnepp W, Büche D, et al. Voluntary stopping of eating and drinking in Swiss outpatient care. *GeroPsych* 2021c; 34: 73–81.
17. [15] Stängle S, Schnepp W, Büche D, et al. Family physicians' perspective on voluntary stopping of eating and drinking: a cross-sectional study. *J Int Med Res* 2020a; 48: 1–15.

18. [16] Stängle S, Schnepp W, Büche D, et al. Long-term care nurses' attitudes and the incidence of voluntary stopping of eating and drinking: A cross-sectional study. *J Adv Nurs* 2020b; 76: 526–34.
19. [68] Gerson SM, Preston NJ and Bingley AF. Medical Aid in Dying, Hastened Death, and Suicide: A Qualitative Study of Hospice Professionals' Experiences From Washington State. *J Pain Symptom Manage* 2020; 59: 679–86 e1.
20. [61] Gerson SM. Hospice professionals' experiences with patients: A qualitative study of suicide and hastened death in Washington State. Dissertation 2018. Lancaster University (United Kingdom).
21. [14] Shinjo T, Morita T, Kiuchi D, et al. Japanese physicians' experiences of terminally ill patients voluntarily stopping eating and drinking: a national survey. *BMJ Support Palliat Care* 2019; 9: 143–45.
22. [65] Stängle S, Schnepp W, Fringer A. The need to distinguish between different forms of oral nutrition refusal and different forms of voluntary stopping of eating and drinking. *Palliat Care Soc Pract* 2019a; 13: 1–7.
23. [69] Saladin N, Schnepp W and Fringer A. Voluntary stopping of eating and drinking (VSED) as an unknown challenge in a long-term care institution: an embedded single case study. *BMC Nurs* 2018; 17: 39.
24. [70] Malpas PJ and Mitchell K. "Doctors Shouldn't Underestimate the Power that they Have": NZ Doctors on the Care of the Dying Patient. *Am J Hosp Palliat Care* 2017; 34: 301–07.
25. [10] Bolt EE, Hagens M, Willems D, et al. Primary care patients hastening death by voluntarily stopping eating and drinking. Ann Fam Med 2015; 13: 421–28.
26. [13] Hoekstra NL, Strack M and Simon A. Bewertung des freiwilligen Verzichts auf Nahrung und Flüssigkeit durch palliativmedizinisch und hausärztlich tätige Ärztinnen und Ärzte - Ergebnisse einer empirischen Umfrage (n=255). [Physicians' attitudes on voluntary refusal of food and fluids to hasten death - Results of an empirical study among 255 physicians]. Z Palliativmed 2015; 16: 68–73.
27. [62] Hoekstra NL. Freiwilliger Verzicht auf Nahrung und Flüssigkeit – eine Bewertung aus Sicht von palliativmedizinischen und hausärztlich tätigen Ärztinnen und Ärzten. [Voluntary Refusal of Food and Fluids – Attitudes from the perspective of palliative care and primary care physicians]. Dissertation 2020, University of Göttingen (Germany).
